# Genetic diversity of next generation antimalarial targets: A baseline for drug resistance surveillance programmes

**DOI:** 10.1016/j.ijpddr.2017.03.001

**Published:** 2017-03-09

**Authors:** Ana Rita Gomes, Matt Ravenhall, Ernest Diez Benavente, Arthur Talman, Colin Sutherland, Cally Roper, Taane G. Clark, Susana Campino

**Affiliations:** a Faculty of Infectious and Tropical Diseases, London School of Hygiene and Tropical Medicine, London, UK; b Wellcome Trust Sanger Institute, Hinxton Cambridge, UK; c Faculty of Epidemiology and Population Health, London School of Hygiene and Tropical Medicine, London, UK

**Keywords:** Antimalarial drugs, Drug-resistant mutations, Gene targets, Genetic diversity

## Abstract

Drug resistance is a recurrent problem in the fight against malaria. Genetic and epidemiological surveillance of antimalarial resistant parasite alleles is crucial to guide drug therapies and clinical management. New antimalarial compounds are currently at various stages of clinical trials and regulatory evaluation. Using ∼2000 *Plasmodium falciparum* genome sequences, we investigated the genetic diversity of eleven gene-targets of promising antimalarial compounds and assessed their potential efficiency across malaria endemic regions. We determined if the *loci* are under selection prior to the introduction of new drugs and established a baseline of genetic variance, including potential resistant alleles, for future surveillance programmes.

## Highlights

• Genetic diversity of 11gene-targets of new antimalarial compounds was investigated.
• Genetic data was analysed for 18 malaria endemic countries.
• No signals of selective pressure was observed.
• A mutation linked to resistance to a new antimalarial was found in African parasites.
• Amino-acid changes close to reported antimalarial resistant mutations were detected.

## Graphical abstract

**Figure:**
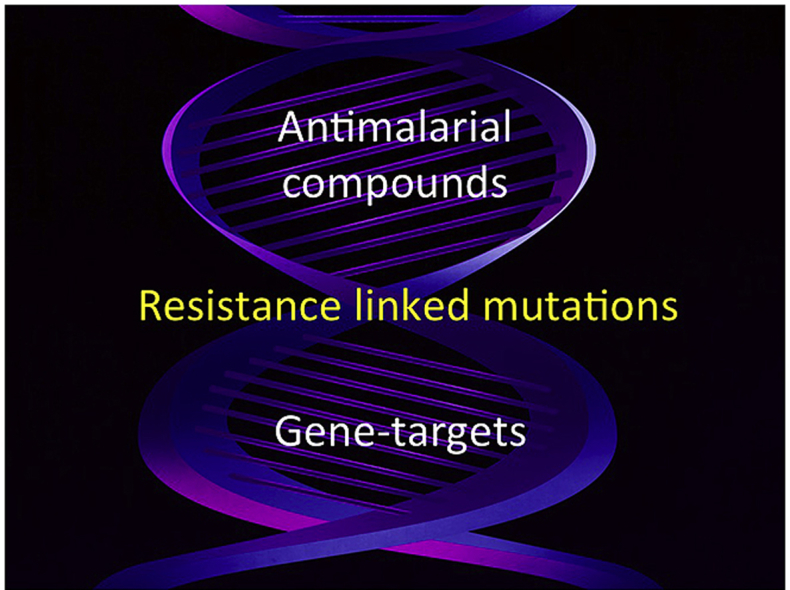

## 1 Introduction

The continuous emergence and spread of resistance to first line antimalarial treatments, including artemisinin and its derivatives, threatens global efforts to reduce the burden of malaria. The development of a fully effective vaccine has been hampered by the complex life cycle of the malaria parasite and the high genetic diversity of key parasite antigens. Thus, antimalarial drugs, particularly those targeting basic cellular machinery common to all stages of the parasite life cycle, are the most promising approaches to control malaria.

The pipeline of antimalarial drugs has greatly expanded over the past decade, particularly because of the strong public-private partnerships and significant investment in innovative technologies (Flannery et al., 2013, Wells et al., 2015). A set of next generation antimalarial compounds, for which the molecular targets are known or being investigated, are currently at various phases of preclinical and clinical assessment (Wells et al., 2015).

Knowledge of parasite molecular drug targets can be exploited to monitor the potential emergence and spread of resistant alleles, particularly from the introduction of a drug, and rapidly inform local policies to tailor interventions. Without knowledge of antimalarial gene targets, the identification and surveillance of resistant alleles needs to be based on accurate clinical drug efficacy trials and genome-wide population genetic studies of field collected samples (Anderson et al., 2011). This approach can be both costly and labour intensive. Alternatively, a powerful strategy to identify mutations linked to resistance, prior to the licensing of a drug, is the use of laboratory-adapted strains to induce selection *in vitro* with sub-lethal and increasing concentrations of drugs. This strategy has led to the identification of polymorphisms in the *Plasmodium (P) falciparum kelch13* gene underlying resistance to artemisinin (Ariey et al., 2014). This gene was confirmed subsequently in association studies in field collected samples (Miotto et al., 2015) and using a reverse genetics approach (Ghorbal et al., 2014).

Here we consider eleven gene-targets of key investigated compounds that due to their efficiency might become the next antimalarial drugs, and for which mutations conferring resistance have been identified in *in vitro* studies (Baragaña et al., 2015, Dong et al., 2011, Flannery et al., 2015, Herman et al., 2015, Kato et al., 2016, LaMonte et al., 2016, Lim et al., 2016, McNamara et al., 2013, Ross et al., 2014). These 11 genes were also selected because they are gene-targets for a range of new antimalarial compounds already under evaluation in clinical trials. We present a survey of the natural genetic variation (SNPs, insertions and deletions (indels), copy number variants (CNVs)) and diversity in these gene-targets using a publicly available global collection of ∼2000 *P. falciparum* “field” parasite genomes from 18 countries. We use the variation to establish whether these regions are already under selective pressure, and report a baseline reference to assist future surveillance programmes with observing emergence of resistance mutations.

## 2 Materials and methods

Eleven gene-targets were analysed: *PF3D7_1113300 (Pfugt), PF3D7_1036800 (Pfact), PF3D7_0109800 (PfcPheRs), PF3D7_0603300 (Pfdhodh), PF3D7_0509800 (Pfpi4k), PF3D7_0321900 (Pfcarl), PF3D7_1211900 (Pfatp4), PF3D7_1451100 (PfeEF2), PF3D7_1320600 (Pfrab11A), PF3D7_1213800 (Pfprs)* and *Mal_Mito_3 (PfCYTB)* (see Table 1). Genome variation data was analysed for isolates from East Africa (Kenya, Tanzania, n = 35), West Africa (Burkina Faso, The Gambia, Ghana, Guinea, Mali, Nigeria, n = 521), Central Africa (Democratic Republic of Congo (DRC), n = 56), South America (Colombia, Peru, n = 24), South Asia (Bangladesh, n = 53) and Southeast Asia (Cambodia, Laos, Myanmar, Papua New Guinea, Thailand, Vietnam, n = 1187).

**Table 1:** Drug targets and genetic polymorphisms.

Gene-target ID	Gene	Active Compounds	Drug development stage	SNP (non-synonymous)	Non-reference allele frequency >5% (non-synonymous)	Known antimalarial resistant mutations
*PfcPheRs (PF3D7_0109800)*	Phenylalanine-tRNA ligase alpha subunit	Bicyclic azetidine	Nonclinical development	44 (28)	5 (3)	L550V
*Pfcarl (PF3D7_0321900)*	Cyclic amine resistance locus protein	Imidazolopiperazines, benzimidazolyl piperidines	Clinical trials	152 (90)	34 (21)	0
*Pfpi4k (PF3D7_0509800)*	Phosphatidylinositol-4 kinase	Imidazopyrazines, aminopyridine class, quinoxaline, 2-aminopyradines	Clinical trials	182 (129)	62 (49)	0
*Pfdhodh (PF3D7_0603300)*	Ddihydroorotate dehydrogenase	triazolopyrimidine-based inhibitor, N-alkyl-5-thiophene-2-carboxamides	Clinical trials	50 (26)	8 (3)	0
*Pfact (PF3D7_1036800)*	Acetyl-CoA transporter	Imidazolopiperazines	Clinical trials	46 (22)	13 (6)	0
*Pfugt (PF3D7_1113300)*	UDP-galactose transporter	Imidazolopiperazines	Clinical trials	30 (12)	10 (6)	0
*Pfatp4 (PF3D7_1211900)*	P-type cation transporting ATPase	Spiroindolones, sulfonamide, carboxamide, pyrazoles,dihydroisoquinolones	Clinical trials	123 (75)	34 (23)	0
*Pfprs (PF3D7_1213800)*	Proline-tRNA synthetase	Febrifugine and derivates	Nonclinical development	66 (31)	16 (9)	0
*Pfrab11A (PF3D7_1320600)*	Ras-related protein Rab-11A	Aminopyridine class	Clinical trials	5 (1)	0 (0)	0
*PfeEF2 (PF3D7_1451100)*	Elongation factor 2	Quinoline-4-carboxamide (DDD107498)	Preclinical development	34 (1)	4 (0)	0
*PfCYTB (mal_mito_3)*	Cytocrome b	Atovaquone, tetracyclic benzothiazepine, benzylsulfonamide, decoquinate	Clinical drug, clinical trial	46 (9)	7 (2)	0

Sequencing data was generated by the Pf3k project (www.malariagen.net/pf3k), is open access and is described in (Miotto et al., 2015). Whole genome analysis of these data has also been recently described (Ravenhall et al., 2016) and we used a set of characterised high quality SNPs and indels identified in the 11 candidate target genes. In addition, larger structural variants (e.g. CNVs) in these regions were identified using *Delly* software (Rausch et al., 2012). Using the SNP variants, population genetic analyses were performed to establish if targeted coding regions are under selection. In particular, the Tajima's D method was applied to detect regions under balancing selection (R package Pegas); extended haplotype homozygosity approaches (|iHS|, XP-EHH) were applied to identify long-range positive directional selection, and F_ST_ statistics were used to assess population differentiation (see (Ravenhall et al., 2016) for a detailed description of these methods).

## 3 Results

Across the eleven gene-targets, a total of 778 SNPs were identified, with half (n = 424, 54.5%) leading to non-synonymous changes **(**Table 1, Supplementary Table 1). The overall genetic diversity was low, with the majority of SNPs (75.1%) having minor allele frequencies of less than 5%. The SNP density (number of SNPs per kbp) across genes was similar (∼1 SNP per 33bp), except for those coding for the ras-related protein (*Rab11A*, 1 SNP per 258.6bp), elongation factor 2 (*eEF2*, 1 SNP per 73.4bp) and the acetyl-CoA transporter (*ACT,* 1 SNP per 64.2bp), all with lower density, suggesting greater gene conservation. The *pfact* gene was recently identified to be the target, together with the UDP-galactose transporter gene-target (*Pfugt),* of a variety of imidazolopiperazine compounds. One of these compounds (KAF156) has potent activity against gametocytes and parasite liver stages, and is currently in Phase II clinical trial (Lim et al., 2016). Rab11A is a molecular target for aminopyridine class compounds (McNamara et al., 2013), and eEF2 is the target for quinoline-4-carboxamide (DDD107498) compounds, both with activity against multiple lifecycle parasite stages (Baragaña et al., 2015). The eEF2 protein mediates GTP-dependent translocation of the ribosome along the mRNA and is required during protein synthesis. The Rab11A protein is likely involved in cytokinesis and interacts with another antimalarial gene-target, the *Pfpi4k* (McNamara et al., 2013). Only one non-synonymous SNP was detected for each of these two genes, supporting their likely essential function. A low number of non-synonymous SNPs (19.6%) was also detected for the mitochondrial cytochrome *b* (*MtcytB*) gene. This gene is the target for several antimalarial compounds under evaluation (Dong et al., 2011) and atovoquone, a longstanding antimalarial drug used in combination with proguanil in Malarone™ for the curative and prophylactic treatment of malaria.

The *Pfpi4k* gene has the highest percentage of non-synonymous SNPs (71.4%), and is a lipid kinase that is a cellular target of imidazopyrazines and quinoxaline compounds (McNamara et al., 2013). This gene probably acts in the Golgi complex and regulates essential membrane trafficking events (McNamara et al., 2013). We also detected a high number of non-synonymous SNPs for the *PfcPhers* (62.7%), *Pfatp4* (60.9%) and *Pfcarl* (59.2%) genes. The *Pfatp4* and *Pfcarl* have been extensively studied as antimalarial targets. The *Pfcarl* is an uncharacterized protein-coding gene that also localises in the Golgi apparatus of the parasite and the *Pfatp4* locus probably functions as a Na^+^-efflux ATPase (Flannery et al., 2015, LaMonte et al., 2016). Several mutations in these genes have previously been reported, particularly for *Pfatp4*, to confer resistance to a growing number of antimalarial compounds that are structurally unrelated (Table 2**)**. None of these mutations have been identified in the set of global field isolates considered here. However, several non-synonymous mutations were observed in their vicinity (Table 2**)**. The *PfcPhers* is a recently discovered gene-target that can be inhibited by a novel compound (bicyclic azetidine BRD3444) with action in all parasite life stages (liver, blood, and transmission), and with the advantage that can act in a single low-dose (Kato et al., 2016). One of the non-synonymous SNPs identified for this gene in the global dataset is a mutation (L550V amino-acid change) linked to *in vitro* resistance to BRD3444 (Kato et al., 2016). This mutation was detected in a few field isolates from the Democratic Republic of Congo (frequency 1.79%) and Ghana (0.5%) (Table 2). We also detected synonymous SNPs in a codon for which an amino-acid change (V545I) has also been implicated in resistance to BRD3444.

**Table 2:** Genetic polymorphisms in target-genes at and surrounding resistant linked mutations.

Gene	Amino-acid substitution^a^	Frequency (%)	Most frequent Population
*PfcPheRs (PF3D7_0109800)*	M316I	0	
	T318A	0.47	Bangladesh, Thailand
	G512E	0	
	K519	0.76, 0.96, 2.68	Cambodia, Vietnam, Laos
	V545I		
	V545	2.6, 5.5	DRC, Kenya
	L550V	1.79, 0.5	DRC, Ghana
	L552	1.1	Guinea
*Pfpi4k (PF3D7_0509800)*	D1311	0.26	
	S1320L	0	
	E1355	0.49	Ghana
	Y1356F	0	
	L1479	0.49	Ghana
	H1484Y	0	
*Pfdhodh (PF3D7_0603300)*	L172F	0	
	E182D	0	
	F188L	0	
	T256	1.052, 0.4	Guinea, Malawi
	I263F	0	
	F227I	0	
	L515F	0.24, 0.09	Ghana, Thailand
	L527I	0	
	L531F	0	
	M536I	0.96, 0.19	Laos, Cambodia
Pfcarl (PF3D7_0321900)	Q821	0.49	Ghana
	P822L	0	
	L830V	0	
	E834K	0	
	S1076N/I	0	
	D1101G	1.54	Ghana
	A1102	0.24	Ghana
	V1103L	0	
	A1135D	0.49	Ghana
	L1136P	0	
	I1139K	0	
*Pfatp4 (PF3D7_1211900)*	Q172H	0	
	V178I		
	A185S	0.49	Ghana
	I203L		
	V204L		
	S312P	0	
	S315	0.02	Malawi
	L350H	0	
	I379N	0	
	I398F	0	
	V400A	0	
	V414D	0	
	T416N	0	
	T418N	0	
	A421L	0	
	P412L	0	
	E895K	0	
	F917L	0	
	L938I	0	
	P966A	0	
	A967G	0	
	K988R	0.45	Malawi
	P990R	0	
	A1158V		
	I1205L	0.18, 0.05, 0.5	Gambia, Malawi, Tanzania
	A1207V		
	T1208S	0.11	Guinea
	L1242	2.67	DRC
	D1247Y	0	
*Pfcprs (PF3D7_1213800)*	L482H	0	
	T1445A	0	
	C1444T	0	
*Pfrab11A (PF3D7_1320600)*	F128	0.96, 0.53	Laos,Vietnam
	D139Y	0	
*PfeEF2 (PF3D7_1451100)*	E134D	0	
	Y185N	0	
	L755F	0	
	T184I	0	
	I185T	0	
	E336G	0	
	S473R	0	
	A481T	0	
	P756A	0	
	L757F	0	
Pfcytb (Mal_Mito_3)	G33A	0	
	Y126C	0	
	G131S	0	
	M133I	0	
	L144S	0	
	V150I	0.22	Ghana
	V234I	0	
	V248I	0.1, 12.5	PNG, Nigeria
	F267V	0	
	V284L	0	
*Pfact (PF3D7_1036800)*	A94T	0	
	R108K	0	
	S110R	0	
	D165N	0	
	S169T	0	
	I190L	2.9, 4.7, 6.7, 3.2, 2.9, 6.7	Bangladesh, Myamar, Thailand, Vietnam, Cambodia, Laos
	C193	0	
	S242	0	
	L253	0	
	G559R	0	
*pfugt (PF3D7_1113300)*	F37V	0	

a In grey are amino-acid changes or silent mutations linked to drug resistance *in vitro*.

Although we did not find reported antimalarial resistance mutations in any of the other genes in the set of clinical isolates, we detected some mutations in their vicinity, including several less than 2 amino-acids away (Table 2**)**. The potential effect of these natural genetic variants on resistance to antimalarial new components should be investigated.

We also assessed the presence of indels and CNVs, as structural variants have been found to be associated with drug resistance in antimalarial treatments, including *pfmdr1* for mefloquine and *pfgch1* for sulfadoxine/pyrimethamine (Ravenhall et al., 2016). Copy number variation in the gene-targets *Pfatp4*, *Pfdhodh,* coding for the enzyme dihydroorotate dehydrogenase (Ross et al., 2014), and *Pfpi4k*, have also been identified in parasite lines resistant to antimalarial compounds. No CNVs were identified across the unique regions of the eleven candidate genes. Indels in both homo-polymeric and tandem repetitive regions were detected, none changing the reading-frame of the respective proteins.

**Fig. 1:**
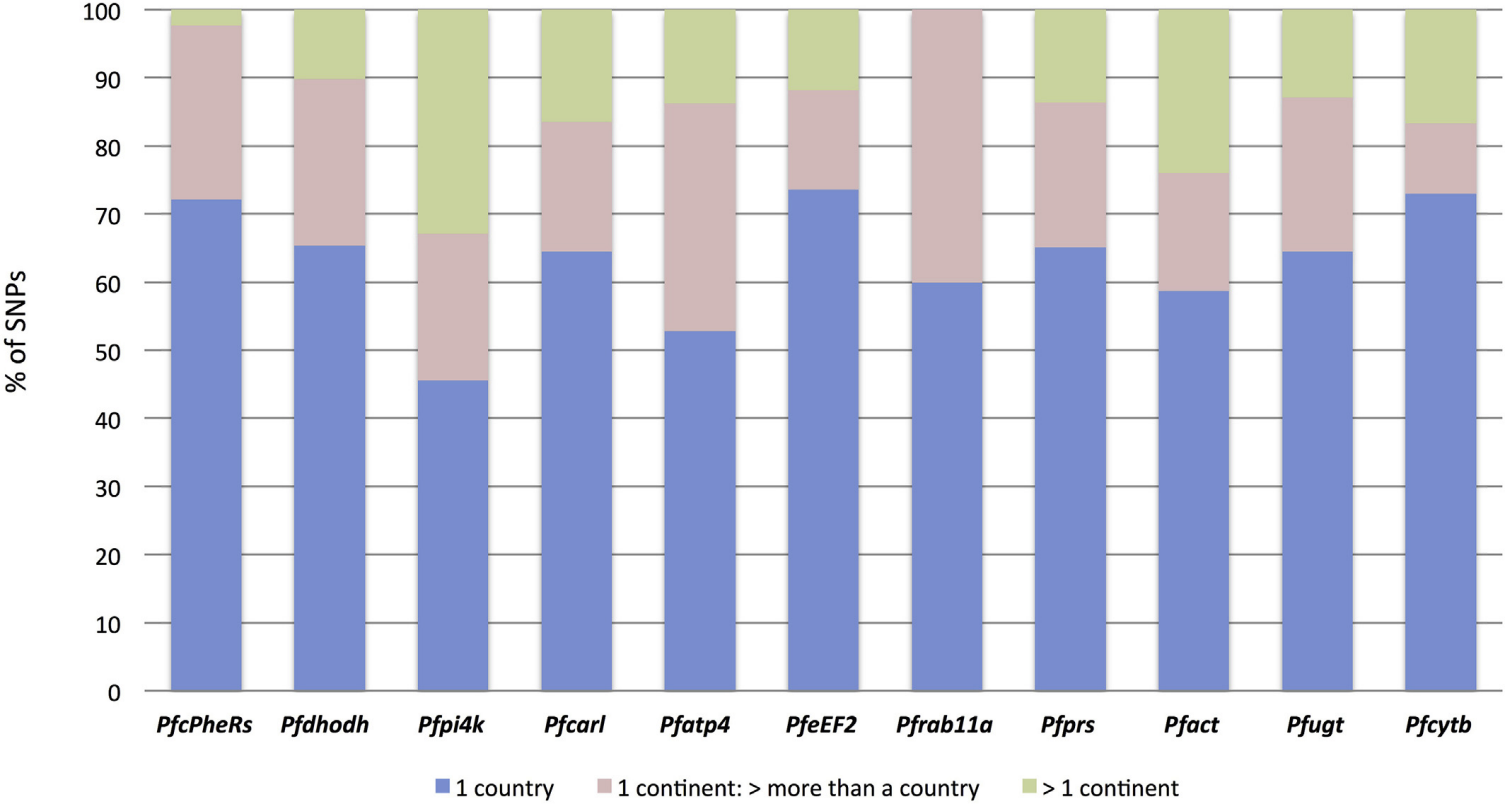
**Geographic distribution of SNPs across eleven gene-targets**. Distribution of genes across countries and continents showed that the majority of SNPs identified were found in only one country.

**Fig. 2:**
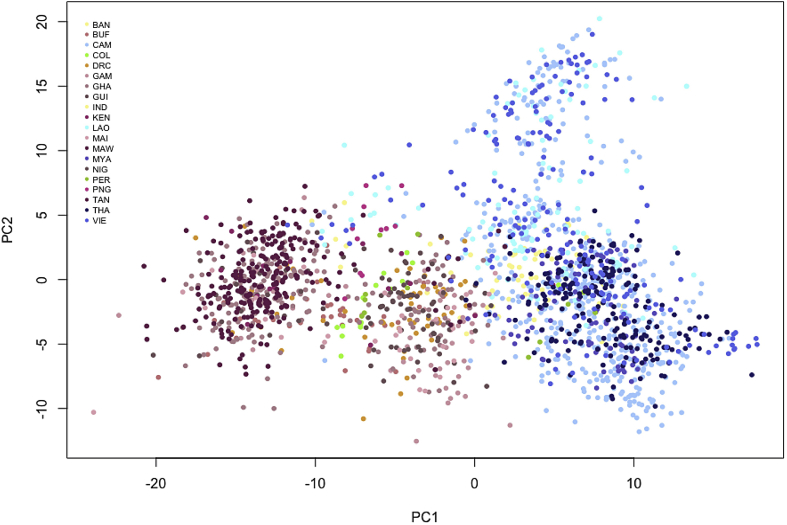
**Population structure at a continental level**. Principal Components (PC) Analysis plot (x axis represents PC1and y axis PC2) on the ∼, 2000 Principal Components (PC) Analysis plot (x axis represents PC1, and y axis PC2) on the ∼2000 P. falciparum field samples from 18 countries and using 778 SNPs identified in the eleven genes-targets.

We also investigated if any of the gene-targets was under selective pressure. Tajima's D values were predominantly negative (82.7%, median −0.32 range −3.51–1.17) indicating an excess of rare alleles, consistent with a historical population expansion of *P. falciparum* and in keeping with results from genome-wide analyses (Ravenhall et al., 2016). There was no evidence of positive directional selection (all |iHS|<2; median = −0.23, min = −1.58, max = 1.65). There was little evidence of selective pressure in the candidate regions, implying that they are likely to be evolving randomly and under neutrality across geographical regions. We detected several SNPs (63.2%) specific to a single country (Fig. 1) or continent (22.7%). Using the 778 SNPs, a principal component analysis revealed clustering by continent (Fig. 2). The *Pfatp4*, *Pfcarl* and *Pfpi4k* genes contributed the most to the observed regional clustering (Supplementary Fig. 1). The F_ST_ measure was used to identify SNPs with allele frequency differences between countries and continents. This analysis revealed nine SNPs with F_ST_ > 0.45, with clear geographic allelic frequency differences (Supplementary Table 2), particularly differentiating African from Asian origins. These SNPs were localized in the *Pfatp4*, *Pfcarl*, *Pfpi4k* and the *Pfcprs* genes. The *Pfcprs i*s a cytoplasmic prolyl-tRNA synthetase and a functional target of febrifugine and its synthetic derivatives with activity at erythrocytic and liver stages (Herman et al., 2015).

## 4 Discussion

Continuous monitoring of drug efficacy and genome selection pressure is crucial to ensure early detection and appropriate response to the emergence of drug resistance. We assessed eleven potential antimalarial gene-targets of compounds that are at various stages of testing, and for which mutations linked to resistance are known. The availability of whole genome sequencing data for worldwide field isolates enabled us to survey the genetic diversity in these targets. We identified one mutation associated with *in vitro* resistance to the antimalarial compounds in low frequency in two African countries. We also identified several amino-acid changes in close proximity to resistance-linked mutations (8 non-synonymous substitution detected <2 amino-acids away). These and other mutations detected in these genes might have a role in the development of resistance, highlighting the need for drug screening with field isolates in addition to laboratory adapted strains. The high divergence of *Plasmodium* biology and lack of crystallized protein structures hindered the assessment of the potential impact of the polymorphisms mutations detected in these genes.

The genetic diversity described here may have a role upon onset of selection, and should be taken into account by surveillance programmes. From these observations, we speculate that for new antimalarial compounds acting on the *PfcPhers* gene, acquired resistance may occur more rapidly in the field, as pre-existing resistant alleles already circulate, although in low frequency, in clinical isolates. Thus, the identification of suitable partner drugs will be crucial to protect its efficacy. This has been effective in prolonging the use of some antimalarial drugs (e.g. atovaquone, artemisinin), which despite resistance readily evolving *in vitro* and in the field, have been used as effective antimalarials in combination therapies.

For the *Pfrab11A* and *Pfe*EF2 gene-targets the particularly low genetic diversity and detection of only 1 non-synonymous mutation could suggest that new antimalarials targeting these genes may have a longer lifespan in the field. Nevertheless, as antimalarial resistant mutations have arisen *in vitro* in all these gene-targets, and despite several produced low fitness mutants that might not survive in the human body and not be transmitted, combination therapies should be considered to increase the useful therapeutic life of these new compounds.

With the continuous emergence of resistance to artemisinin derivatives, the introduction of new antimalarial drugs is urgent and a priori knowledge of the parasite diversity that these drugs are likely to encounter will aid drug resistance monitoring programmes. Overall, the genetic information described here for eleven gene-targets and across 18 countries from malaria endemic regions, forms a baseline diversity that can assist genetic surveillance studies with detecting allele frequency changes associated with the pressure imposed by a newly introduced drug.

## Financial support

This work was supported by the Medical Research Council UK (Grant no. MC_PC_15103 to A.R.G., Grant no. MR/K000551/1, MR/M01360X/1, MR/N010469/1 to T.G.C. and S.C.) and by the Biotechnology and Biological Sciences Research Council (Grant Number BB/J014567/1 to M.R.).

## Conflict of interests

The authors declare no conflict of interest.

## Figures and Tables

**suplefig1:**
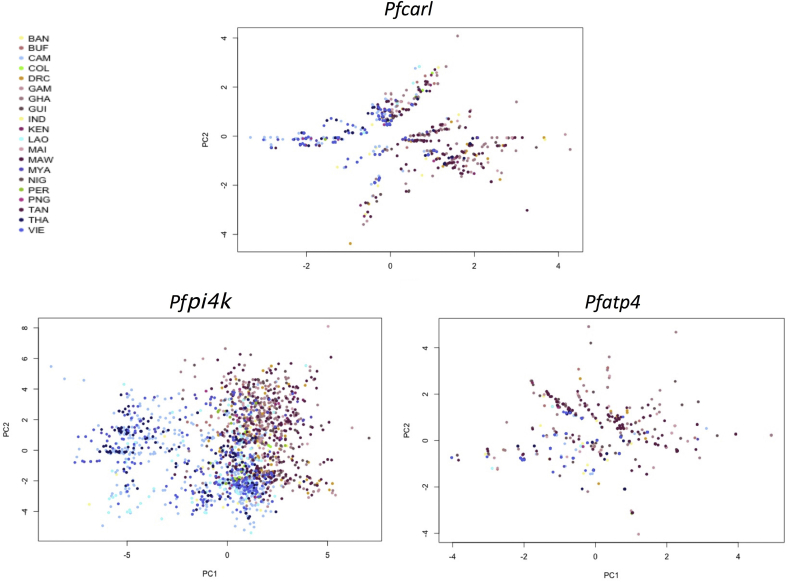
Population structure at a continental level for the *Pfcarl*, *Pfpi4k* and *Pfatp4* genes. Principal Components (PC) Analysis plot (x axis represents PC1, and y axis PC2) on the ∼2000 *P. falciparum* field samples from 18 countries for each gene.

## References

[bib1] Anderson T., Nkhoma S., Ecker A., Fidock D. (2011). How can we identify parasite genes that underlie antimalarial drug resistance?. Pharmacogenomics.

[bib2] Ariey F., Witkowski B., Amaratunga C., Beghain J., Langlois A.-C., Khim N., Kim S., Duru V., Bouchier C., Ma L., Lim P., Leang R., Duong S., Sreng S., Suon S., Chuor C.M., Bout D.M., Ménard S., Rogers W.O., Genton B., Fandeur T., Miotto O., Ringwald P., Le Bras J., Berry A., Barale J.-C., Fairhurst R.M., Benoit-Vical F., Mercereau-Puijalon O., Ménard D. (2014). A molecular marker of artemisinin-resistant Plasmodium falciparum malaria. Nature.

[bib3] Baragaña B., Hallyburton I., Lee M.C.S., Norcross N.R., Grimaldi R., Otto T.D., Proto W.R., Blagborough A.M., Meister S., Wirjanata G., Ruecker A., Upton L.M., Abraham T.S., Almeida M.J., Pradhan A., Porzelle A., Martínez M.S., Bolscher J.M., Woodland A., Norval S., Zuccotto F., Thomas J., Simeons F., Stojanovski L., Osuna-Cabello M., Brock P.M., Churcher T.S., Sala K.A., Zakutansky S.E., Jiménez-Díaz M.B., Sanz L.M., Riley J., Basak R., Campbell M., Avery V.M., Sauerwein R.W., Dechering K.J., Noviyanti R., Campo B., Frearson J.A., Angulo-Barturen I., Ferrer-Bazaga S., Gamo F.J., Wyatt P.G., Leroy D., Siegl P., Delves M.J., Kyle D.E., Wittlin S., Marfurt J., Price R.N., Sinden R.E., Winzeler E.A., Charman S.A., Bebrevska L., Gray D.W., Campbell S., Fairlamb A.H., Willis P.A., Rayner J.C., Fidock D.A., Read K.D., Gilbert I.H. (2015). A novel multiple-stage antimalarial agent that inhibits protein synthesis. Nature.

[bib4] Dong C.K., Urgaonkar S., Cortese J.F., Gamo F.-J., Garcia-Bustos J.F., Lafuente M.J., Patel V., Ross L., Coleman B.I., Derbyshire E.R., Clish C.B., Serrano A.E., Cromwell M., Barker R.H., Dvorin J.D., Duraisingh M.T., Wirth D.F., Clardy J., Mazitschek R. (2011). Identification and validation of tetracyclic benzothiazepines as Plasmodium falciparum cytochrome bc1 inhibitors. Chem. Biol..

[bib5] Flannery E.L., Chatterjee A.K., Winzeler E.A. (2013). Antimalarial drug discovery — approaches and progress towards new medicines. Nat. Rev. Microbiol..

[bib6] Flannery E.L., McNamara C.W., Kim S.W., Kato T.S., Li F., Teng C.H., Gagaring K., Manary M.J., Barboa R., Meister S., Kuhen K., Vinetz J.M., Chatterjee A.K., Winzeler E.A. (2015). Mutations in the P-Type cation-transporter ATPase 4, PfATP4, mediate resistance to both aminopyrazole and spiroindolone antimalarials. ACS Chem. Biol..

[bib7] Ghorbal M., Gorman M., Macpherson C.R., Martins R.M., Scherf A., Lopez-Rubio J.-J. (2014). Genome editing in the human malaria parasite Plasmodium falciparum using the CRISPR-Cas9 system. Nat. Biotechnol..

[bib8] Herman J.D., Pepper L.R., Cortese J.F., Estiu G., Galinsky K., Zuzarte-Luis V., Derbyshire E.R., Ribacke U., Lukens A.K., Santos S.A., Patel V., Clish C.B., Sullivan W.J., Zhou H., Bopp S.E., Schimmel P., Lindquist S., Clardy J., Mota M.M., Keller T.L., Whitman M., Wiest O., Wirth D.F., Mazitschek R. (2015). The cytoplasmic prolyl-tRNA synthetase of the malaria parasite is a dual-stage target of febrifugine and its analogs. Sci. Transl. Med..

[bib9] Kato N., Comer E., Sakata-Kato T., Sharma A., Sharma M., Maetani M., Bastien J., Brancucci N.M., Bittker J.A., Corey V., Clarke D., Derbyshire E.R., Dornan G.L., Duffy S., Eckley S., Itoe M.A., Koolen K.M.J., Lewis T.A., Lui P.S., Lukens A.K., Lund E., March S., Meibalan E., Meier B.C., McPhail J.A., Mitasev B., Moss E.L., Sayes M., Van Gessel Y., Wawer M.J., Yoshinaga T., Zeeman A., Avery V.M., Bhatia S.N., Burke J.E., Catteruccia F., Clardy J.C., Clemons P.A., Dechering K.J., Duvall J.R., Foley M.A., Gusovsky F., Kocken C.H.M., Marti M., Morningstar M.L., Munoz B., Neafsey D.E., Sharma A., Winzeler E.A., Wirth D.F., Scherer C.A., Schreiber S.L. (2016). Diversity-oriented synthesis yields novel multistage antimalarial inhibitors. Nature.

[bib10] LaMonte G., Lim M.Y.-X., Wree M., Reimer C., Nachon M., Corey V., Gedeck P., Plouffe D., Du A., Figueroa N., Yeung B., Bifani P., Winzeler E.A. (2016). Mutations in the Plasmodium falciparum cyclic amine resistance locus (PfCARL) confer multidrug resistance. MBio.

[bib11] Lim M.Y.-X., LaMonte G., Lee M.C.S., Reimer C., Tan B.H., Corey V., Tjahjadi B.F., Chua A., Nachon M., Wintjens R., Gedeck P., Malleret B., Renia L., Bonamy G.M.C., Ho P.C.-L., Yeung B.K.S., Chow E.D., Lim L., Fidock D.A., Diagana T.T., Winzeler E.A., Bifani P. (2016). UDP-galactose and acetyl-CoA transporters as Plasmodium multidrug resistance genes. Nat. Microbiol..

[bib12] McNamara C.W., Lee M.C.S., Lim C.S., Lim S.H., Roland J., Nagle A., Simon O., Yeung B.K.S., Chatterjee A.K., McCormack S.L., Manary M.J., Zeeman A., Dechering K.J., Kumar T.R.S., Henrich P.P., Gagaring K., Ibanez M., Kato N., Kuhen K.L., Fischli C., Rottmann M., Plouffe D.M., Bursulaya B., Meister S., Rameh L., Trappe J., Haasen D., Timmerman M., Sauerwein R.W., Suwanarusk R., Russell B., Renia L., Nosten F., Tully D.C., Kocken C.H.M., Glynne R.J., Bodenreider C., Fidock D.A., Diagana T.T., Winzeler E.A. (2013). Targeting Plasmodium PI(4)K to eliminate malaria. Nature.

[bib13] Miotto O., Amato R., Ashley E.A., MacInnis B., Almagro-Garcia J., Amaratunga C., Lim P., Mead D., Oyola S.O., Dhorda M., Imwong M., Woodrow C., Manske M., Stalker J., Drury E., Campino S., Amenga-Etego L., Thanh T.-N.N., Tran H.T., Ringwald P., Bethell D., Nosten F., Phyo A.P., Pukrittayakamee S., Chotivanich K., Chuor C.M., Nguon C., Suon S., Sreng S., Newton P.N., Mayxay M., Khanthavong M., Hongvanthong B., Htut Y., Han K.T., Kyaw M.P., Faiz M.A., Fanello C.I., Onyamboko M., Mokuolu O.A., Jacob C.G., Takala-Harrison S., Plowe C.V., Day N.P., Dondorp A.M., Spencer C.C.A., McVean G., Fairhurst R.M., White N.J., Kwiatkowski D.P. (2015). Genetic architecture of artemisinin-resistant Plasmodium falciparum. Nat. Genet..

[bib14] Rausch T., Zichner T., Schlattl A., Stütz A.M., Benes V., Korbel J.O. (2012). DELLY: structural variant discovery by integrated paired-end and split-read analysis. Bioinformatics.

[bib15] Ravenhall M., Benavente E.D., Mipando M., Jensen A.T.R., Sutherland C.J., Roper C., Sepúlveda N., Kwiatkowski D.P., Montgomery J., Phiri K.S., Terlouw A., Craig A., Campino S., Ocholla H., Clark T.G. (2016). Characterizing the impact of sustained sulfadoxine/pyrimethamine use upon the Plasmodium falciparum population in malawi. Malar. J..

[bib16] Ross L.S., Gamo F.J., Lafuente-Monasterio M.J., Singh O.M.P., Rowland P., Wiegand R.C., Wirth D.F. (2014). *In Vitro* resistance selections for Plasmodium falciparum dihydroorotate dehydrogenase inhibitors give mutants with multiple point mutations in the drug-binding site and altered growth. J. Biol. Chem..

[bib17] Wells T.N.C., van Huijsduijnen R.H., Van Voorhis W.C. (2015). Malaria medicines: a glass half full?. Nat. Rev. Drug Discov..

