# Corrigendum to “Genetic diversity of next generation antimalarial targets: A baseline for drug resistance surveillance programmes” [Int. J. Parasitol. Drugs and Drug Resist. 7 (2017) 174–180]

**DOI:** 10.1016/j.ijpddr.2017.11.001

**Published:** 2017-11-06

**Authors:** Ana Rita Gomes, Matt Ravenhall, Ernest Diez Benavente, Arthur Talman, Colin Sutherland, Cally Roper, Taane G. Clark, Susana Campino

**Affiliations:** a Faculty of Infectious and Tropical Diseases, London School of Hygiene and Tropical Medicine, London, UK; b Wellcome Trust Sanger Institute, Hinxton, Cambridge, UK; c Faculty of Epidemiology and Population Health, London School of Hygiene and Tropical Medicine, London, UK

The authors regret that the reported non-synonymous mutation L550V in the *PfcPhers* gene is a synonymous mutation. We would like to correct this information in the manuscript.

In the Results section, second paragraph, it should read: “The L550V mutation was not identified in the set of global field isolates considered here. At this position we detected a synonymous mutation.” In the Discussion, second paragraph, this sentence can be omitted “From these observations, we speculate that for new antimalarial compounds acting on the *PfcPhers* gene, acquired resistance may occur more rapidly …”. L550V should be omitted from Tables 1 and 2 and Suppl. Table 1.

The authors would like to apologies for any inconvenience caused.

