# Supplementary material for: Genetic diversity of next generation antimalarial targets: A baseline for drug resistance surveillance programmes
Source: Int J Parasitol Drugs Drug Resist. 2017 Mar 9;7(2):174–80. doi: 10.1016/j.ijpddr.2017.03.001 (PMC5379905; doi:10.1016/j.ijpddr.2017.03.001)
Supplement: Supplementary _table 2 [file mmc1.doc]

Supplementary
Table 2	Allele frequencies of genetic polymorphisms with Fst>0.45


	
Gene	Pfprs
PF3D7_1213800	pfatp4
PF3D7_1211900	Pfcarl
PF3D7_0321900	Pfpi4k
PF3D7_0509800	
Genomic position (bp) Amino-acid
Continent/ country	substitution	592329

V36L	529559	532131	529418

Q1081K	Syn	G1128R	924915	924907	923803

Q605L	SYN	L234F	413414	413426

D1049E	D1053E	
S. America	PER COL	0.00
0.00	58.3	100.0	100.0
60.0	40.0	73.3	66.7	100.0	41.7
26.7	26.7	33.3	29.2	54.2
16.7	56.7	
West Africa                        GUI GAM MAI BUF GHA
NIG	0.00
0.00
0.00
0.00
0.25
0.00	48.4	16.8	47.4
64.5	18.2	69.1
50.0	14.3	54.3
43.6	16.7	35.9
39.1	17.8	39.4
37.5	25.0	25.0	12.1	81.6	52.6
15.5	86.4	50.9
5.7	67.1	48.6
3.8	73.1	51.3
6.7	71.8	56.7
12.5	100.0	62.5	6.9	60.1
7.7	62.5
14.3	51.4
19.2	61.5
12.7	55.9
0.0	62.5	
Central Africa	DRC	0.00	58.0	12.5	60.7	7.1	70.5	62.5	15.2	69.4	
East Africa		TAN MAW
KEN	0.00
0.45
0.00	61.1	13.9	63.9
48.6	10.2	48.6
29.4	17.6	47.1	16.7	83.3	58.3
8.6	78.1	52.7
29.4	64.7	70.6	22.2	67.6
18.7	63.5
5.9	53.1	
South Asia	BAN	63.89	9.3	64.8	26.9	43.5	86.1	31.5	65.2	52.4	
South East Asia                    MYA THA CAM LAO
VIE	27.37
28.81
71.06
74.52
68.98	1.1	77.9	31.6
2.9	72.1	45.7
0.9	52.8	65.3
3.4	60.6	26.4
0.0	69.5	36.6	44.2	90.0	24.7
48.1	95.5	17.1
43.6	87.2	22.6
51.9	94.7	6.7
48.4	97.9	33.6	61.3	23.6
57.6	13.2
64.4	19.9
72.5	32.6
69.9	31.4	
Melanesia	PNG	9.09	9.1	13.6	13.6	50.0	100.0	18.2	66.7	60.0	
